# IL-12-armed oncolytic HSV-2 enhances CAR T cell efficacy against pancreatic cancer in xenografted models

**DOI:** 10.3389/fimmu.2025.1664289

**Published:** 2026-01-16

**Authors:** Chongfeng Xu, Jian Wu, Weikang Liu, Xiaoya Zhou, Qian Liang, Guoya Li, Yang Wang, Yanliang Liu, Qiying Cai, Zilong Tang, Chunyi Tu, Han Hu, Binlei Liu, Shufang Meng

**Affiliations:** 1 Cell Collection and Research Center, Institute of Biological Products, National Institutes for Food and Drug Control (NIFDC), Beijing, China; 2 State Key Laboratory of Drug Regulatory Science, Beijing, China; 3 Beijing Key Laboratory of Quality Control and Non-clinical Research and Evaluation for Cellular and Gene Therapy Medicinal Products, Beijing, China; 4 Key Laboratory of the Ministry of Health for Research on Quality and Standardization of Biotech Products, Beijing, China; 5 School of Life and Health Sciences, Hubei University of Technology, Wuhan, China; 6 Department of Respiratory Zhucheng People’s Hospital, Shandong, China; 7 Wuhan Binhui Biotechnology Co. Ltd., Wuhan, China

**Keywords:** CAR-T, HSV-2, IL-12, mesothelin, oncolytic virus, pancreatic cancer

## Abstract

**Introduction:**

Chimeric antigen receptor (CAR) T cells show limited efficacy in solid tumors. Oncolytic viruses (OVs), especially those expressing immunomodulatory cytokines like interleukin-12 (IL-12), potentiate to synergize with CAR-T therapy.

**Methods:**

We integrated an IL-12-expressing oncolytic herpes simplex virus type 2 (oHSV-2-IL-12) with mesothelin-targeting SS1-ICOSBBZ-CAR-T to treat Capan-2 pancreatic cancer cells xenografts in B-NDG immunodeficient mice.

**Results:**

SS1-ICOSBBZ-CAR-T alone exhibited partial anti-tumor activity, but could not eradicate established tumors. Intra-tumoral oHSV-2-IL-12 administration potently enhanced CAR-T efficacy, achieving complete and durable tumor elimination even at reduced CAR-T doses. After the initial tumors were fully eliminated by combination therapy, mice were re-challenged by inoculating mesothelin-negative and mesothelin-positive tumor cell lines on the left and right flanks, respectively. In the combination treatment group, mesothelin-positive tumors failed to form new tumors within two weeks after re-challenge, whereas mesothelin-negative tumors grew normally. These findings indicate that oHSV-2-IL-12 combined with CAR-T therapy confers durable, antigen-specific protection against tumor re-challenge. Mechanistically, oHSV-2-IL-12 promoted CAR-T proliferation and persistence in peripheral blood and spleen. IL-12 expression also augmented the direct oncolytic effect of oHSV-2 in immunodeficient hosts.

**Discussion:**

This synergistic approach achieves durable potent tumor clearance with reduced CAR-T doses, offering a transformative strategy against pancreatic cancer and other challenging solid malignancies.

## 1 Introduction

Pancreatic cancer remains one of the most lethal malignancies worldwide, characterized by rapid progression, and poor response to current therapeutic regimens. Despite advances in surgery, chemotherapy, and radiotherapy, the prognosis for patients with advanced pancreatic cancer remains dismal. The incidence of pancreatic cancer is rising steadily, mortality remains high, and effective treatment options are scarce—underscoring an urgent need for more powerful therapeutic strategies.

Chimeric antigen receptor (CAR)-T cell therapy has shown remarkable success in hematological malignancies, however, its efficacy in solid tumors, including pancreatic cancer, remains limited. Among the targets explored in CAR-T therapy, mesothelin (MSLN) is one of the most extensively studied for solid tumors. However, the therapeutic efficacy of MSLN-targeted CAR-T cell has been limited in clinical trials for pancreatic cancer, often resulting in temporary stable disease as the best response (1, 2) even treated with other immunotherapy drug (3). To enhance the efficacy of CAR-T, third-generation CARs incorporating both ICOS and 4-1BB co-stimulatory domains (ICOSBBZ), fourth-generation cytokines armed CAR-T have been developed and exhibit improved T-cell activity and persistence (4, 5). Despite these improvements, studies indicate that mesothelin-targeted CAR-T therapy failed to achieve complete tumor eradication even in animal models, making combination therapies a crucial strategy.

Oncolytic viruses (OVs) offer a dual antitumor action: directly lysing malignant cells while reshaping the tumor microenvironment to enhance immune responses. Among these, herpes simplex virus type 2 (HSV-2) stands out for its tumor-selectivity, genetic engineering flexibility, and minimal pre-existing immunity. Despite their multifaceted mechanisms, OVs alone often fall short in efficacy (6). To boost the therapeutic potential of HSV-2, researchers have engineered it to deliver immunostimulatory cytokines—particularly interleukin-12 (IL-12). IL-12 is a potent pro-inflammatory cytokine that enhances T cell proliferation, cytotoxic activity, and cytokine production—qualities beneficial for tumor control (7, 8). However, systemic IL-12 administration is limited by severe toxicity, making localized, tumor-restricted delivery via oncolytic vectors an attractive alternative. While HSV-2-based OVs have shown promise in clinical trials (9, 10), the potential of an IL-12-expressing oncolytic HSV-2 (oHSV2-IL-12) to augment the efficacy of mesothelin-targeted CAR-T therapy, specifically SS1-ICOSBBZ-CAR T cells, remains unexplored (5, 11, 12). Given the documented synergistic benefits between OVs and CAR T cells (13–15), alongside promising preliminary evidence for oHSV-2-driven IL-12 delivery (16), we hypothesize that oHSV2-IL-12 will significantly enhance the antitumor activity of SS1-ICOSBBZ-CAR-T cells against refractory pancreatic cancer. This study is designed to test this novel combination strategy (17, 18).

In this study, we investigated the therapeutic potential of combining an IL-12-armed oncolytic HSV-2 (oHSV-2-IL-12) with mesothelin-targeted SS1-ICOSBBZ-CAR-T cells against subcutaneous Capan-2 pancreatic cancer xenografts established in immunodeficient B-NDG mice. Specifically, we sought to determine whether localized intratumoral delivery of IL-12 via oHSV-2-IL-12 could enhance the antitumor efficacy of SS1-ICOSBBZ-CAR-T cells and elucidate the underlying mechanisms. This work directly addresses the critical, yet unexplored, potential of leveraging oHSV-2-IL-12 to potentiate CAR-T cell therapy in pancreatic cancer, offering a novel strategy to overcome the limitations of monotherapies and advance the treatment of solid tumors.

## 2 Materials and methods

This study was carried out in strict accordance with the recommendations in the Guide for the Care and Use of Laboratory Animals of the National Institutes of Health (NIH). The animal experiments were approved by the Institutional Animal Care and Use Committee (IACUC) of the National Institutes for Food and Drug Control (NIFDC), (China Animal Quarantine Certificate (Welfare) No. 2023(B) 047). All efforts were made to minimize animal suffering, and humane euthanasia procedures anesthesia.

### 2.1 Cell lines and oHSV-2 viruses

Vero, SKOV3 (human ovarian), Panc-1, and Capan-2 cell lines were obtained from ATCC. Luciferase-expressing lines (Capan-2-luc, Panc-1-luc) were created via lentiviral transduction followed by puromycin selection (3 µg/mL) and clonal isolation. Panc-1-luc-MSLN cells were similarly engineered to express human mesothelin and validated by flow cytometry. All lines tested negative for mycoplasma and were STR-authenticated. Culture conditions: Capan-2-luc and SKOV3 in McCoy’s 5A + 10% FBS (with puromycin for Capan-2-luc); Panc-1-luc in DMEM + 10% FBS (with puromycin); Vero in MEM + 10% FBS; incubated at 37 °C and 5% CO_2_. Oncolytic HSV-2 vectors (oHSV-2-GFP and oHSV-2-IL-12) derived from HG52 strain with ICP34.5 deletion and IL-12 insertion were produced, purified, and titered by plaque assay by Binhui Biotech (Wuhan, China) (16).

### 2.2 CAR-expressing lentiviral vectors

A third-generation, four-plasmid lentiviral system (pMDLg/pRRE, pRSV-Rev, pCMV-VSV-G, pRRLSIN backbone) was used. The backbone promoter was switched to EF-1α. Chimeric cDNA sequences containing SS1-TM ICOS–ICOS-BBz were custom synthesized (Nanjing GenScript Biotechnology Co., Ltd, China), digested with BamHI and SalI and ligated into the pRRLSIN-EF-1a-MCS. Plasmids were sequence-verified. Lentivirus production and titration were performed in 293T cells by Huaxia Yingtai (Beijing).

### 2.3 Isolation, transduction, expansion of primary human T lymphocytes and CAR-T manufacture

PBMCs were isolated from healthy donor apheresis samples (Shanghai Miaoshun Biotechnology Co., Ltd.). CD4^+^ and CD8^+^ T cells were purified using negative selection (Miltenyi, Cat# 130-096-535), activated with TransAct (Miltenyi Biotec; Cat# 130-111-160) at a density of 1 × 10^6^ cells per 10 μL reagent for 24 hours (24 h), and cultured in TexMACS + IL-15 (84 U/mL) and IL-7 (500 U/mL). After 24 h, cells were transduced at multiplicities of infection (MOI) = 3 and maintained with cytokine support. CAR-T cells were harvested after 12 days.

### 2.4 Cytotoxicity *in vitro* using a luciferase-based assay

Target cells (Capan-2-luc, Panc-1-luc, Panc-1-luc-MSLN) were plated in 96-well format at respective densities in McCoy’s or DMEM + 10% FBS. After 24 h, CAR-T cells were added at various E:T ratios. Triton X-100 (1%) served as a positive lysis control. After 30 h co-culture, Bright-Glo luciferase reagent (Promega, Cat# E2620) was added and luminescence measured using a GloMax^®^ 96 Microplate Luminometer (Promega) with a 1-second integration time at 560 nm. Percent cytotoxicity was calculated as:


Cytotoxicity=[1−(X−Min)/( Max− Min)]×100%

X = experimental RLU, Max = effector-untreated control, Min = Triton-X 100-treated control.

### 2.5 Dynamic assessment of oHSV-2 oncolytic activity measured via real-time cell analysis

Capan-2-luc cells (5×10^4^/well) were seeded in xCELLigence E-plates (Agilent, Cat# 300600890), then infected with oHSV-2-IL-12 at MOIs of 0.05, 0.5, or 5. After 2 h adsorption, medium with 2.5% FBS was added. Cell index (CI) was monitored every 10 min for 96 h. In the same real-time cytolysis assay, KT_80_ (time to reach 80% cytolysis) was also calculated.

### 2.6 Detection of IL-12 expression by enzyme-linked immunosorbent assay

*In vitro*, Capan-2-luc cells (5×10^5^–6×10^5^/well) were infected with oHSV-2-IL-12 (MOI 0.1 or 0.5). Cell and supernatant samples were collected at 24 and 48 h post-infection. IL-12 p70 levels were measured using RayBio^®^ Human IL-12 p70 ELISA Kit (Cat# ELH-IL12P70-1). *In vivo*, tumor-bearing B-NDG mice received three intratumoral doses of oHSV-2-IL-12. Tumors were harvested on day 9 post-first dose, homogenized, lysed, and IL-12 quantified via Abcam p70 kit Human IL-12 p70 SimpleStep ELISA^®^ Kit (abcam, ab223592), normalized to tissue weight.

### 2.7 Animal models

Immunodeficient B-NDG (NOD.Cg-PrkdcscidIl2rgtm1/Bcgen) female mice (18–21 g, SPF) from Biocytogen Pharmaceuticals (Beijing) Co., Ltd were housed at NIFDC vivarium. All procedures were IACUC-approved.

### 2.8 Pancreatic cancer xenograft tumor model of in immunodeficient mice

2×10^6^ Capan-2-luc cells were injected subcutaneously (s.c.). Tumor volume (V) was measured by calipers:. V = 1/2 × L × W × W. Endpoint: volume ≥ 1,000 mm³.

### 2.9 *In vivo* bioluminescence imaging

Mice received intraperitoneal D-luciferin (150 mg/kg; PerkinElmer, Cat#122799) and were imaged using IVIS Spectrum Imaging System (PerkinElmer)10 min post-injection. Bioluminescence signals were quantified using Living Image software (PerkinElmer) and expressed as total flux (photons/second) within a region of interest drawn around the tumor site.

### 2.10 *In vivo* treatments

*In vivo* xenograft tumors were established by s.c. injection of 2 × 10^6^ Capan-2 cells in PBS. On day 10 (tumor size: 50–100 mm³), mice received 1×10^7^ CAR-T cells intravenously in CAR-T monotherapy experiments. In combination therapy, mice received 1×10^7^ CAR-T cells intravenously and intratumoral injections of oHSV-2-IL-12 or oHSV-2-GFP (1×10^5^ CCID_50_ in 50 µL PBS) every 3 days for 3 doses. For OV monotherapy dose titration, oHSV-2-IL-12 was administered at 1×10^6^, 1×10^5^, or 1×10^4^ CCID_50_. Except for CAR-T monotherapy, where Capan-2 is treated 10 days after inoculation, OV therapies or OV combination with CAR-T therapies are administered 14 days after Capan-2 inoculation. Peripheral blood was obtained from retro-orbital bleeding. Blood and spleen were sampled for CAR-T persistence (human CD45^+^CD3^+^CAR^+^) by flow cytometry.

### 2.11 Tumor rechallenge

Pancreatic cancer–xenografted B-NDG mice that achieved complete remission (≥ 14 days tumor-free) were re-challenged s.c. with 2×10^6^ Capan-2-luc (right flank) and 2×10^6^ Panc-1-luc (left flank). Tumor growth and bioluminescence were monitored weekly.

### 2.12 Sample collection and tissue processing

Blood: 70 μL collected via retro-orbital bleed on days 7, 9, and every 3 days until day 27 post-treatment.7Tumors: harvested on treatment day 9 post-first oHSV-2-IL-12 dose. Homogenized (Shanghai Jingxin Industrial Development Co., Ltd., Shanghai, China) in PBS-lysis buffer (absin, Cat# abs9225) with protease inhibitors (absin, Cat# abs9162); lysates centrifuged and supernatants retained.Spleens: harvested, processed through 70 μm strainers, RBC-lysed with BioLegend buffer (BioLegend, Cat# 420302), and used for flow cytometry.

### 2.13 Flow cytometric analysis

The following fluorescent reagents were used for staining: MSLN-PE (ACRO, Cat# MSN-HP2H5) for CAR, CD4-AmCyan (BD, Cat# 562970), and CD8-APC-Cy7 (BD, Cat# 557834), each diluted at 1:100; CD3-FITC (BD, Cat# 555332) diluted at 1:10; and 7-AAD (BD, Cat# 559925).

CAR-T Phenotyping: Cells were stained in PBS + 2% FBS with antibodies against CD3, CD4, CD8, viability dye 7-AAD, and CAR detected via PE-labeled mesothelin. 7-AAD unstained live cells were gated, and CD3^+^ cells were identified. Within the CD3^+^ population, CAR^+^ (PE postive) T cells were further gated, and the proportions of CD4^+^ and CD8^+^ cells within the CAR^+^ T cell population were analyzed.

CAR+ T cell monitoring *In Vivo*: Blood and spleen cells stained similarly, blood was processed with BD FACS™ Lysing Solution. Tumor homogenates were stained with human CD45-BV650, MSLN-PE, CD3, and 7-AAD. Analysis was performed on an LSRFortessa cytometer (Becton Dickinson) using FlowJo. Live CD45+ cells were gated, the proportion of CAR^+^ cells within the CD3^+^ population was analyzed.

### 2.14 Single-cell cytokine profiling

After 24 h recovery, SS1-ICOSBBZ-CAR-T cells were co-cultured with Capan-2 target cells at E:T = 2:1 for 48 h. CD4^+^ and CD8^+^ subsets were separated using the human CD8 MicroBeads (Miltenyi), stained with AF647-conjugated antibodies (IsoPlexis,STAIN-1002–1 and STAIN-1003-1), loaded onto IsoLight chips (IsoPlexis), and analyzed using IsoSpeak v3.0.1.

### 2.15 Statistical analysis

Analyses conducted in GraphPad Prism 9.0. Data are mean ± SD. Two-group comparisons were by two-tailed Student’s t−test; multiple groups by one-way ANOVA with Tukey’s range test. Tumor growth was analyzed by two-way repeated measures ANOVA with variance normalization if necessary.

## 3 Results

### 3.1 SS1-ICOSBBZ-CAR-T cells exhibit modest cytotoxicity against pancreatic tumors *in vitro* and *in vivo*

**Figure 1:**
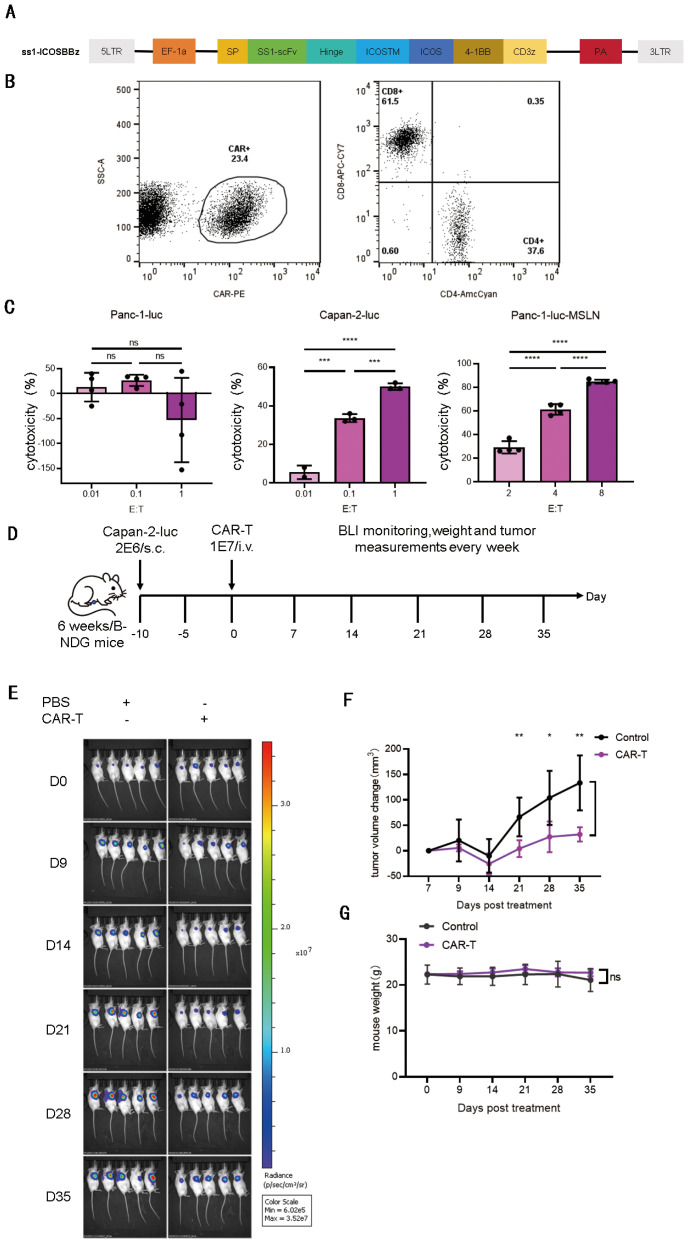
Mesothelin chimeric antigen receptor (CAR)-T cells SS1-ICOSBBZ-CAR-T showed specific and modest anti-tumor capacity *in vitro* and *in vivo*. **(A)** Schematic diagram of the CAR structure. We constructed a third-generation MSLN-targeted CAR-T. This CAR incorporates the single-chain variable fragment (scFv) derived from the SS1 antibody for MSLN recognition, an ICOS-derived transmembrane domain, the intracellular domains contained two co-stimulatory molecules ICOS and 4-1BB, and the T cell receptor ζ (TCR-ζ) signaling domain. **(B)** Percentage of CAR positive T cells at day 12 (left) and CD4+ and CD8+ subsets (right) composition of the final CAR-T cell product. The CAR was stained by human mesothelin protein conjugated to PE. **(C)** Specific cytolytic activity of SS1-ICOSBBZ-CAR-T cells *in vitro* using luciferase assay. CAR-T cells were incubated with tumor cell lines for 30 hours at different E:T ratio. Panc-1-luc (mesothelin negative, left), Capan-2-luc (mesothelin positive, middle), Panc-1-luc-MSLN (mesothelin positive, right). **(D)** Schematic timeline of the antitumor efficacy experiment design. 2 × 10^6^ Capan-2-luc cells were subcutaneously inoculated (s.c.) into the right flank of six-week-old B-NDG mice, tumors were established for 10 days prior to treatment initiation. Then, SS1-ICOSBBz-CAR-T cells or PBS (200 μL control) were administered via tail vein injection, (n = 5). **(E)** Bioluminescence imaging of mice at indicated days after treatment. **(F)** Quantification of tumor burden dynamics following CAR-T therapy. Tumor volume was monitored weekly during 35 days, and tumor volume changes were calculated relative to baseline (Day 0 set as 0% change). The tumor size at day 7 served as the reference for weekly measurements. Statistical significance of volume changes was assessed using t-test (n = 5 per group). **(G)** Body weight of mice treated with SS1-ICOSBBZ-CAR-T cells were measured. All data are presented as means ± standard deviation. *P < 0.05, **P < 0.01.

Inducible T-cell co-stimulator (ICOS), a member of the CD28/CTLA-4 family, plays a pivotal role in modulating immune responses and T-cell differentiation. To harness this property, we engineered third-generation CAR-T cells by transducing peripheral blood T cells from healthy donors with a lentiviral vector encoding the SS1-ICOSBBZ-CAR. This CAR comprises an SS1-derived single-chain variable fragment (scFv) targeting mesothelin, the transmembrane domain of ICOS, and intracellular signaling domains from ICOS, 4-1BB, and CD3ζ. The design of this CAR construct is illustrated in **Figure 1A**, as previously described by Guedan et al. (4). The synthesized CAR gene was cloned into a lentiviral vector, packaged using 293T cells, and subsequently used to transduce peripheral blood T cells, generated SS1-ICOSBBZ-CAR-T cells.

After completing the manufacturing of the CAR-T cells, we evaluated the final product’s quality by assessing the CAR expression (CAR^+^ percentage) and determining the proportions of CD4^+^ and CD8^+^ T cells. Flow cytometric analysis revealed that the CAR expressing rate was 23.4% (**Figure 1B**, left), among CD3^+^ T cells, 37.6% were CD4^+^ and 61.5% were CD8^+^ (**Figure 1B**, right), resulting in a CD4/CD8 ratio of 0.61.

To evaluate the cytotoxic function of SS1-ICOSBBZ-CAR-T cells, *in vitro* co-culture assays with pancreatic cancer cell lines revealed potent, dose-dependent and antigen specific killing of MSLN -positive targets but not MSLN-negative controls (**Figure 1C**). Specifically, the CAR-T cells lysed ~30% of MSLN^+^ Capan-2-luc cells at a 1:10 effector-to-target (E:T) ratio, increasing to 50% at 1:1. similarly, they achieved 20% lysis against MSLN^+^ Panc-1-luc-MSLN cells at 2:1 E:T, escalating to 80% at 8:1 within 24 hours. Critically, no significant cytotoxicity (<5% lysis) was observed against MSLN^-^ Panc-1-luc cells. These results confirm the potent and antigen-specific antitumor activity of SS1-ICOSBBZ-CAR-T cells *in vitro* (**Figure 1C**).

We next evaluated the antitumor efficacy in an immune-deficient B-NDG mouse model bearing subcutaneous pancreatic tumors *in vivo*. Capan-2-luc cells (2×10^6^) were subcutaneously inoculated into the right flank of B-NDG mice to establish tumors. On day 10 post-engraftment, mice received 1×10^7^ SS1-ICOSBBZ-CAR-T cells via tail vein injection (experimental timeline, **Figure 1D**). CAR-T treatment significantly suppressed tumor growth (p<0.01, **Figures 1E, F**), though complete eradication was not achieved. Tumor regrowth emerged from day 28 onward, indicating transient efficacy. Notably, no significant body weight loss occurred during treatment (**Figure 1G**), suggesting minimal systemic toxicity of SS1-ICOSBBZ-CAR-T cells.

### 3.2 OHSV-2-IL-12 demonstrates oncolytic activity against pancreatic cancer cells *in vitro* and *in vivo*

**Figure 2:**
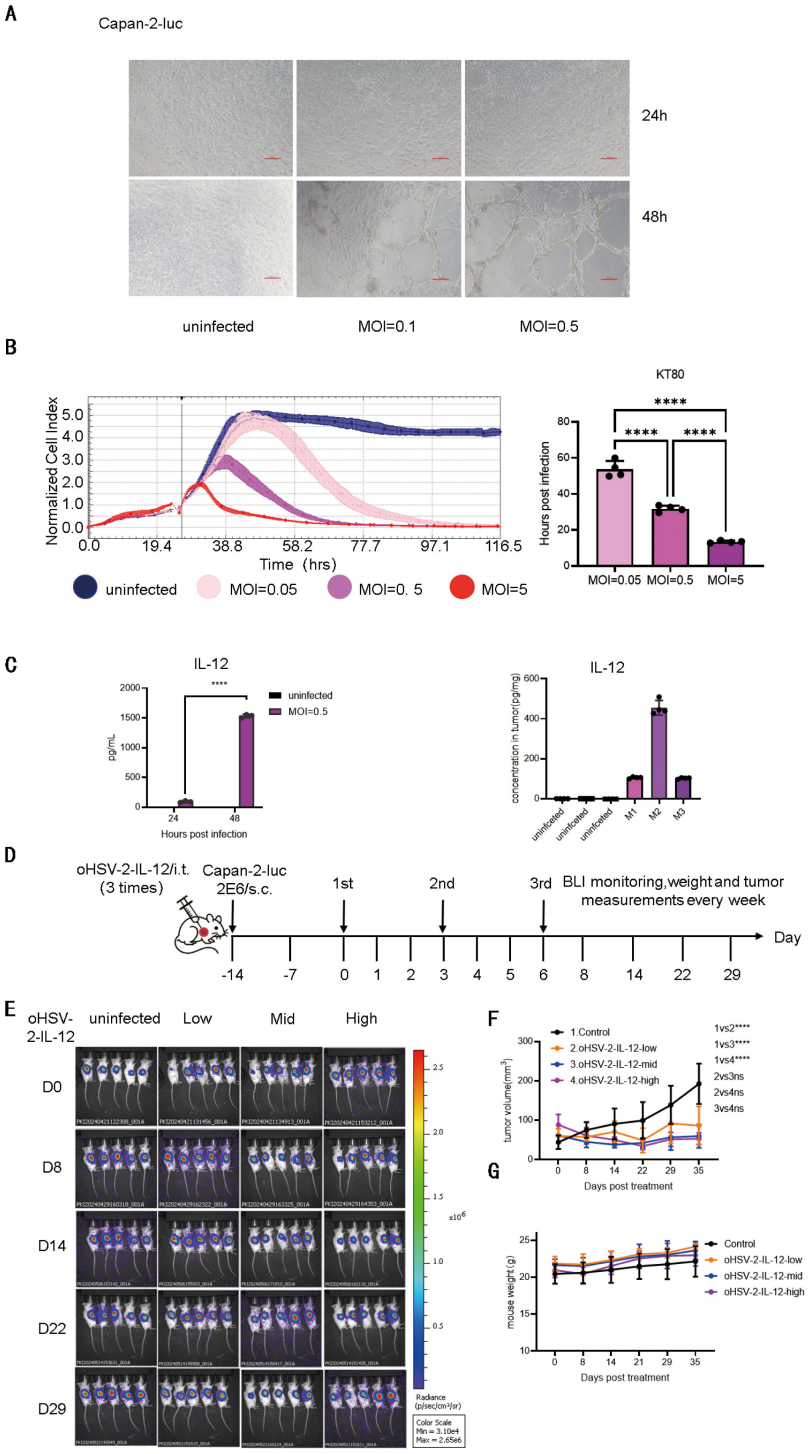
*In vitro* and *in vivo* cytotoxicity effects of oHSV-2-IL-12 on pancreatic cancer cells. **(A)** Capan-2-luc cells were infected with oHSV-2-IL-12 at MOIs of 0.1 and 0.5. Cell morphology was monitored at 24 and 48 hours post-infection using light microscopy. Representative images depict virus-induced cytopathic effects, including cell rounding and detachment, indicative of oncolytic activity. **(B)** Real-time cytolysis assessed by RTCA and determination of KT80 values. Representative RTCA graphs to illustrate cytolytic effects of oHSV-2-IL-12 on Capan-2-luc cells. Capan-2-luc cancer cells were plated in duplicates in RTCA plates and incubated overnight. Thereafter oHSV-2-IL-12 was added at MOI 0.05, 0.5 and 5. The impedance was recorded every 10 min for 96 h. Cell index (CI) values were monitored every 10 minutes for 96 hours using the xCELL igence RTCA system. CI values were normalized to the time point of virus addition. Percent cytolysis was calculated, and the time required to reach 80% cytolysis (KT80) was determined for each MOI (right). **(C)** IL-12 secretion by Capan-2-luc cells infected with oHSV-2-IL-12 *in vitro* and *in vivo*. Capan-2-luc cells were infected with oHSV-2-IL-12 at MOIs of 0.5. After 24 hours and 48 hours, supernatants were collected, and IL-12 levels were measured by ELISA (left). Six-week-old B-NDG mice bearing subcutaneous Capan-2-luc tumors received intratumoral injections of oHSV-2-IL-12 on days 0, 3, and 6. On day 9 post-initial injection, tumors were harvested, homogenized, and IL-12 levels were measured by ELISA (n=3). Statistical significance between the two MOI groups was assessed using an unpaired two-tailed Student’s t-test (n=3). **(D)** Schematic representation of the *in vivo* treatment protocol using oHSV-2-IL-12 in six-week-old B-NDG mice bearing subcutaneous Capan-2-luc tumors. Mice received three injections of oHSV-2-IL-12 (i.t.) at two-day intervals,. Weekly assessments included BLI, body weight measurement, and tumor volume evaluation. **(E)***In vivo* cancer cells BLI results obtained weekly following oHSV-2-IL-12 (i.t.) inoculation. Mice were divided into three dose groups: high (1×10^6^ CCID_50_), medium (1×10^5^ CCID_50_), and low (1×10^4^ CCID_50_). **(F)** Weekly tumor volume measurements of different groups. Tumor response to oHSV-2-IL-12 therapy was analyzed by two-way ANOVA with Tukey’s multiple comparisons (n=5/group). **(G)** Weekly body weight changes during treatment. Statistical significance: **** p <0.0001. ns, not significant.

OVs can infect and lyse tumor cells, and OVs engineered to express cytokines can modulate immune activity—these are crucial antitumor mechanisms of OV. Accordingly, we verified the capability of the oHSV-2 to infect and lyse pancreatic cancer cell lines both *in vitro* and *in vivo*, and the expression of IL-12 within tumors. OHSV-2-IL-12 has been reported to exhibit antitumor activity in the murine CT26 colon cancer model (16). In this study, oHSV-2-IL-12 demonstrated potent, dose-dependent antitumor activity against Capan-2-luc pancreatic cancer cells, successfully infecting cells at MOIs of 0.1 and 0.5 and inducing evident cytopathic effects (CPE) within 24 to 48 hours post-infection (**Figure 2A**). RTCA confirmed this dose-dependent cytolytic activity, showing that higher MOIs caused a faster and more pronounced decrease in Cell Index (CI) (**Figure 2B**, left). The cytotoxicity kinetics, measured as the time required to achieve 80% cell lysis (KT_80_), exhibited a clear inverse relationship with MOI. KT_80_ was approximately 54 hours at an MOI of 0.05, decreased to 34 hours at an MOI of 0.5, and reached about 12 hours at a higher MOI of 5 (**Figure 2B**, right), conclusively demonstrating that increased viral doses accelerate and enhance tumor cell killing.

Subsequent investigations demonstrated that oHSV-2-IL-12 drives functional IL-12 expression both *in vitro* and *in vivo*. *In vitro*, infecting Capan-2-luc cells at MOIs of 0.1 and 0.5 triggered a dose-dependent increase in IL-12 secretion, with ELISA quantifying approximately 1.5 ng/mL in culture supernatants by 48 hours post-infection (**Figure 2C**, left). *In vivo*, intratumoral administration of oHSV-2-IL-12 (three times, days 0, 3 and 6) into Capan-2-luc xenograft-bearing B-NDG mice led to pronounced IL-12 expression in tumor tissues. ELISA analysis of tumor homogenates 9 days post-first treatment revealed IL-12 concentrations of ~100 pg/mg tissue in two mice and ~400 pg/mg in the third (**Figure 2C**, right). Following intratumoral injection of oHSV-2-IL-12, IL-12 was undetectable in serum samples. This confirms that IL-12 expression mediated by the OV was localized specifically to the injected tumor site and sites of viral infection/spread, with no systemic leakage. Collectively, these findings demonstrate that oHSV-2-IL-12 achieves tumor-restricted delivery of IL-12, minimizing systemic exposure and the associated toxicity risks. This highly localized expression profile underscores the favorable safety profile of this approach for targeted immunotherapeutic applications.

We evaluated the *in vivo* antitumor efficacy of oHSV-2-IL-12 by intratumoral administration at three dose levels (1×10^6^, 1×10^5^, 1×10^4^ CCID_50_/mouse) and as shown in **Figure 2D**. BLI results showed that between days 8 and 14, high- and medium-dose groups displayed significantly reduced tumor signal compared to untreated controls, whereas the low-dose group did not (**Figure 2E**). Consistent with these findings, tumor volumes in all treated groups were significantly lower than controls (**Figure 2F**); however, there were no statistically significant differences among the three dose groups. Body weights remained unchanged across all groups (**Figure 2G**), indicating that oHSV-2-IL-12 was well tolerated without detectable systemic toxicity.

Taken together, these data demonstrate that oHSV−2−IL−12 not only infects tumor cells and tissues but also drives IL−12 gene expression within infected cells. Compared to untreated controls, oHSV−2−IL−12 exhibited significant antitumor activity *in vivo*, as evidenced by both imaging and tumor growth measurements. However, although treatment inhibited tumor progression, it did not induce tumor regression or complete remission, indicating that oHSV−2−IL−12 monotherapy is insufficient for robust antitumor efficacy.

### 3.3 OHSV-2-IL-12 enhances the antitumor activity of SS1-ICOSBBZ-CAR-T cells by promoting their expansion and persistence

**Figure 3:**
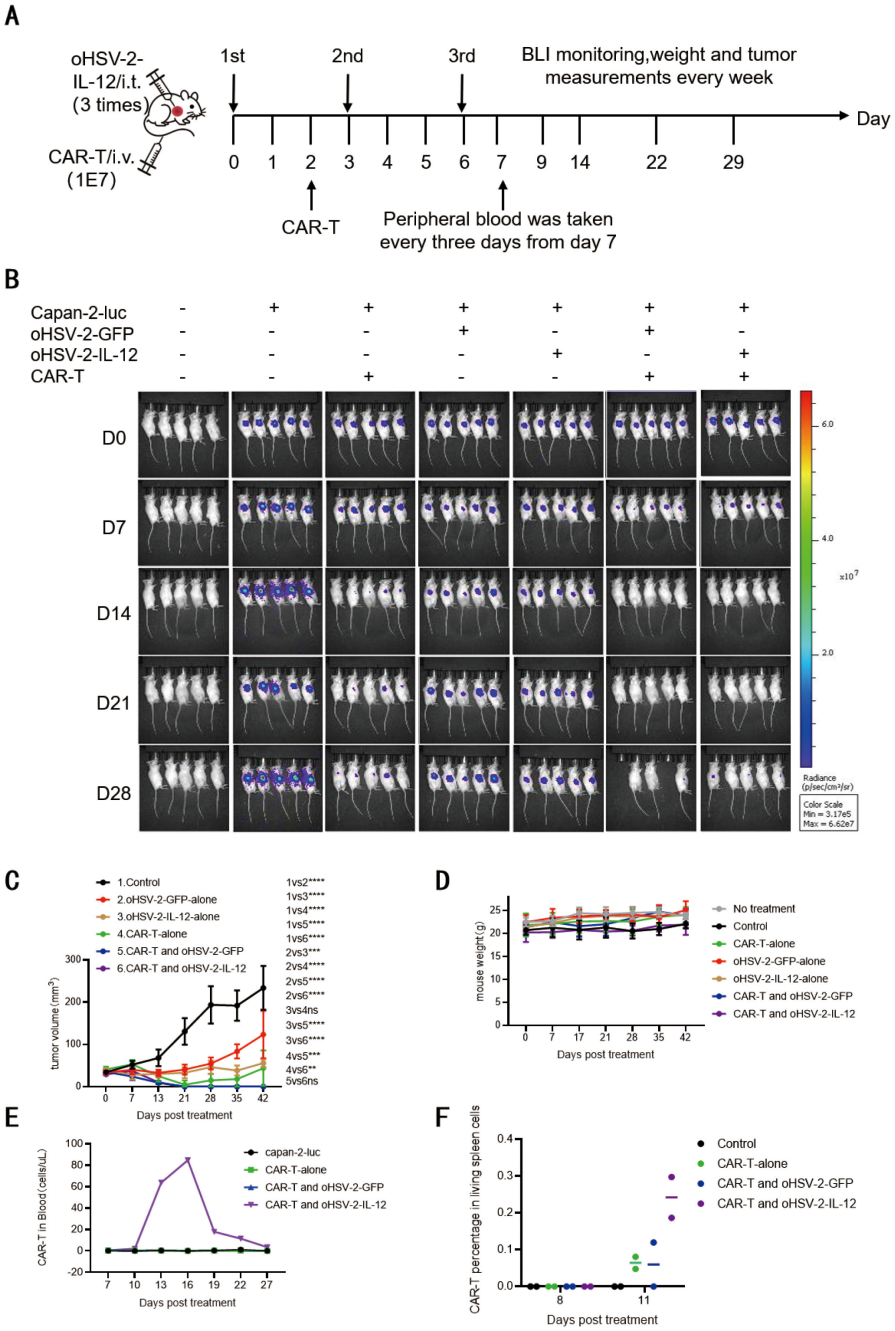
Combination oHSV-2-IL-12 and CAR-T achieved complete tumor clearance by promoting expansion of SS1-ICOSBBZ-CAR-T cells. **(A)** Schematic diagram of the combined oHSV-2-IL-12 and SS1-ICOSBBz-CAR-T treatment regimen in 6-week-old B-NDG mice bearing subcutaneous Capan-2-luc tumors established for 14 days. Xenografted pancreatic tumors were injected i.t. with 100 μL PBS, 1×10^5^ CCID_50_ oHSV-2-GFP, oHSV-2-IL-12 on treatment days 0, 3, and 6. 200 μL PBS or 1×10^7^ SS1-ICOSBBz-CAR-T cells were i.v. in B-NDG mice on treatment day 2. On treatment day 7, retro-orbital venous blood of B-NDG mice were sampled every 3 days. **(B)** Representative tumor BLI measurement. **(C)** Weekly tumor volume measurements were performed. **(D)** Mice were weighed weekly. **(E)** CAR-T cells in mouse retro-orbital venous blood detected by flow cytometry every 3 days starting from treatment day 7. Significance was assessed using two way ANOVA combined with Tukey’s multiple comparisons test (each group n=5). **(F)** CAR-T cells frequency in splenic single-cell suspensions in mice were detected by flow cytometry after sacrificing mice on treatment day 8 and day 11. ns:p>0.05;**p<0.01;***p<0.001;**** p<0.0001.

We hypothesized that oHSV-2-IL-12 plus MSLN targeted CAR-T cells could overcome the incomplete tumor clearance observed with each monotherapy. To evaluate whether combining OV therapy with CAR-T cells enhances tumor killing, we therefore used B-NDG mouse Capan-2-luc xenograft models, where 2×10^6^ tumor cells were implanted subcutaneously. On day 14 post-inoculation, medium-dose oHSV-2-IL-12 (1×10^5^ CCID_50_) was administered intratumorally and repeated every three days for a total of three injections, SS1-ICOSBBZ-CAR-T cells (1×10^7^ cells/mouse) were delivered two days after the first OV injection, the experimental design is illustrated in **Figure 3A**. oHSV-2-GFP served as the control virus.

As hypothesized, the combination therapy significantly outperformed either CAR-T or OV monotherapy (**Figures 3B, C**). Complete tumor elimination occurred on days 14 and 21 post-treatment in the combination groups (**Figure 3B**). Statistical analysis of tumor volumes revealed that OV + CAR-T treatment showed significantly reduced tumor size compared to monotherapy groups, namely, the tumor volume in group 5 (oHSV-2-GFP + CAR-T) was significantly smaller than that in groups 2 (oHSV-2-GFP) (p < 0.0001), group 3 (oHSV-2-IL-12) (p < 0.0001), and group 4 (CAR-T) (p < 0.001), and tumor size in group 6 (oHSV-2-IL-12 + CAR-T) also significantly smaller than those monotherapy groups (**Figure 3C**). However, no significant difference in antitumor efficacy was observed between the two combination groups (5 vs. 6; p > 0.05).

Among three monotherapy groups, CAR-T appeared most potent as shown by bioluminescence imaging (**Figure 3B**); but tumor volume analysis showed no significant difference between CAR-T and oHSV-2-IL-12 groups (group 4 vs. 3, p > 0.05) (**Figure 3C**). In contrast, CAR-T treatment significantly reduced tumor size compared to the oHSV-2-GFP control (group 4 vs. 2, p < 0.0001), likely because oHSV-2-GFP lacks the capacity to fully activate host immune cell mediated cytotoxicity in immunodeficient B-NDG mice. Importantly, tumor volumes were also significantly less in the oHSV-2-IL-12 group than in oHSV-2-GFP (group 3 vs. 2, p < 0.001), demonstrating enhanced antitumor efficacy conferred by the IL-12-armed OV.

OHSV-2-IL-12 monotherapy demonstrated significantly superior antitumor activity over oHSV-2-GFP monotherapy in our study. However, we observed no significant difference in antitumor efficacy between the combination therapy group (CAR-T cells + oHSV-2-IL-12) and the control group (CAR-T cells + oHSV-2-GFP) in the xenograft tumor model. To elucidate whether oHSV-2-IL-12 enhances CAR-T cell therapy, we analyzed CAR-T cell proliferation and persistence in the peripheral blood and spleens of the mice. Peripheral blood was collected every three days starting one week post-treatment from five mice per group. Flow cytometry analysis of CD3^+^CAR^+^ T cells revealed that these cells were detected only in the oHSV-2-IL-12 + CAR-T combination group (**Figure 3E**). Within this group, SS1-ICOSBBZ-CAR-T cells were first detectable on day 13, peaked by day 16, and subsequently declined, becoming nearly undetectable by day 22. Substantial inter-animal heterogeneity in CAR-T cell numbers was observed. **Figure 3E** presents data from a single representative mouse. The exclusive detection of CAR^+^ T cells in the oHSV-2-IL-12 + CAR-T combination group suggests oHSV-2-IL-12 drives SS1-ICOSBBZ-CAR-T cell expansion and persistent.

Consistent with the peripheral blood findings, oHSV-2-IL-12 also increased the number of CAR-T cells in the spleen. At the study endpoint, spleens were harvested, dissociated, and analyzed for CAR-T cells within single-cell suspensions by flow cytometry (**Figure 3F**). On day 11 post-treatment, CD3^+^CAR^+^ cells were detectable in the spleens of all groups receiving CAR-T cell therapy. While oHSV-2-GFP combination therapy resulted in slightly higher CAR-T cell frequencies compared to CAR-T cell monotherapy, the oHSV-2-IL-12 + ICOSBBZ-CAR-T group exhibited the highest CAR-T frequency. These findings suggest that IL-12 augments both the expansion and splenic homing of SS1-ICOSBBZ-CAR-T cells. Thus, anti-MSLN SS1-ICOSBBZ-CAR-T cells expanded in the periphery and migrate to or persist within the spleen more effectively when supported by oHSV-2-IL-12.

### 3.4 *In vivo* combination of oHSV-2-IL-12 and SS1-ICOSBBz-CAR-T confers protection against antigen-specific tumor re-challenge

In the xenograft tumor model, the combination therapy achieved complete tumor clearance. Therefore, we sought to determine whether CAR-T treatment could induce durable immunological protection by tumor rechallenge experiment.

**Figure 4:**
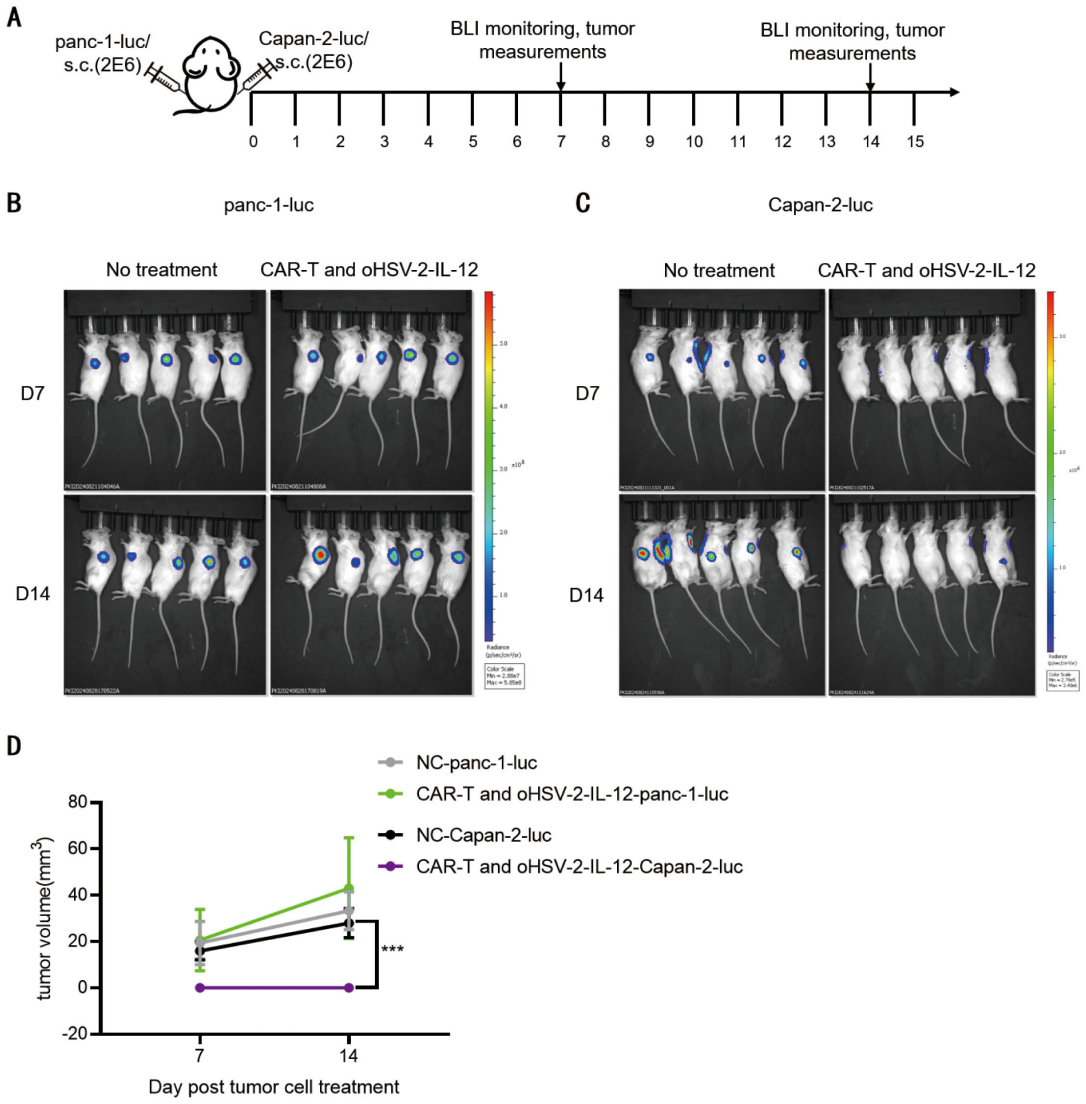
*In vivo* combination oHSV-2-IL-12 and SS1-ICOSBBz-CAR-T prevents antigen-specific tumor rechallenge. **(A)** Schematic of tumor rechallenge in mice receiving combination therapy with oHSV-2-IL-12 and MSLN-ICOSBBz-CAR-T. Following complete tumor clearance within two weeks post-treatment (1×10^5^ oHSV-2-IL-12 + 1×10^5^ SS1-ICOSBBz-CAR-T), mice were rechallenged via bilateral subcutaneous flank injections of 2×10^6^panc-1-luc cells (mesothelin-negative) and 2×10^6^ Capan-2-luc cells. Tumor growth was monitored by weekly BLI and tumor volume measurements. **(B)**Panc-1-luc subcutaneously implanted into the left flank of B-NDG mice after 2 weeks elimination of first Capan-2-luc, tumor growth was monitored by weekly BLI. **(C)** Similarly, a re-challenge was performed by Capan-2-luc cells (S.c.) into the ipsilateral flank of B-NDG mice. Tumor growth was monitored weekly via *in vivo* BLI (right). **(D)** Time-dependent second tumor burden was measured and quantified for indicated experimental mouse groups. Experimental mice were sacrificed when the primary tumor size reaches 1000 mm^3^ (n = 5).

As depicted in **Figure 3**, on day 28 after the combined treatment with 1×10^5^ CCID_50_ oHSV-2-IL-12 and 1×10^7^ SS1-ICOSBBz-CAR-T cells, maintained a tumor-free for two weeks. At this time point, a re-challenge experiment was performed in the same Capan-2-luc xenograft mouse model. The schematic diagram of the re-challenge experimental is shown as **Figure 4A**. Simultaneously, oHSV-2-IL-12 + ICOSBBZ-CAR-T group and the blank group, which had neither received any tumor implantation nor treatment previously, was similarly injected with Capan-2-luc cells on the right side and Panc-1-luc cells on the left side. Tumor development was monitored using BLI and tumor volume was measured on days 7 and 14 post tumor cell injection. The results demonstrated that in the combination therapy group, the reintroduced Capan-2-luc cells failed to form new tumors, whereas tumors developed at the Panc-1-luc cells planted side. In the untreated blank group, tumors developed on both sides, as shown in **Figures 4B, C**. These results indicate that the combination therapy maintained tumor-free status for at least 5 weeks from the initial CAR-T administration and prevented tumor formation for over 2 weeks after rechallenge (**Figure 4D**). These results suggest that the combined therapy induced a durable, tumor-specific immune response capable of preventing tumor recurrence following the combination therapy of oHSV-2-IL-12 and SS1-ICOSBBz-CAR-T cells.

### 3.5 Unveiling the therapeutic role of oHSV-2-IL-12 in combination therapy with CAR-T by reducing the dose of SS1-ICOSBBZ-CAR-T

**Figure 5:**
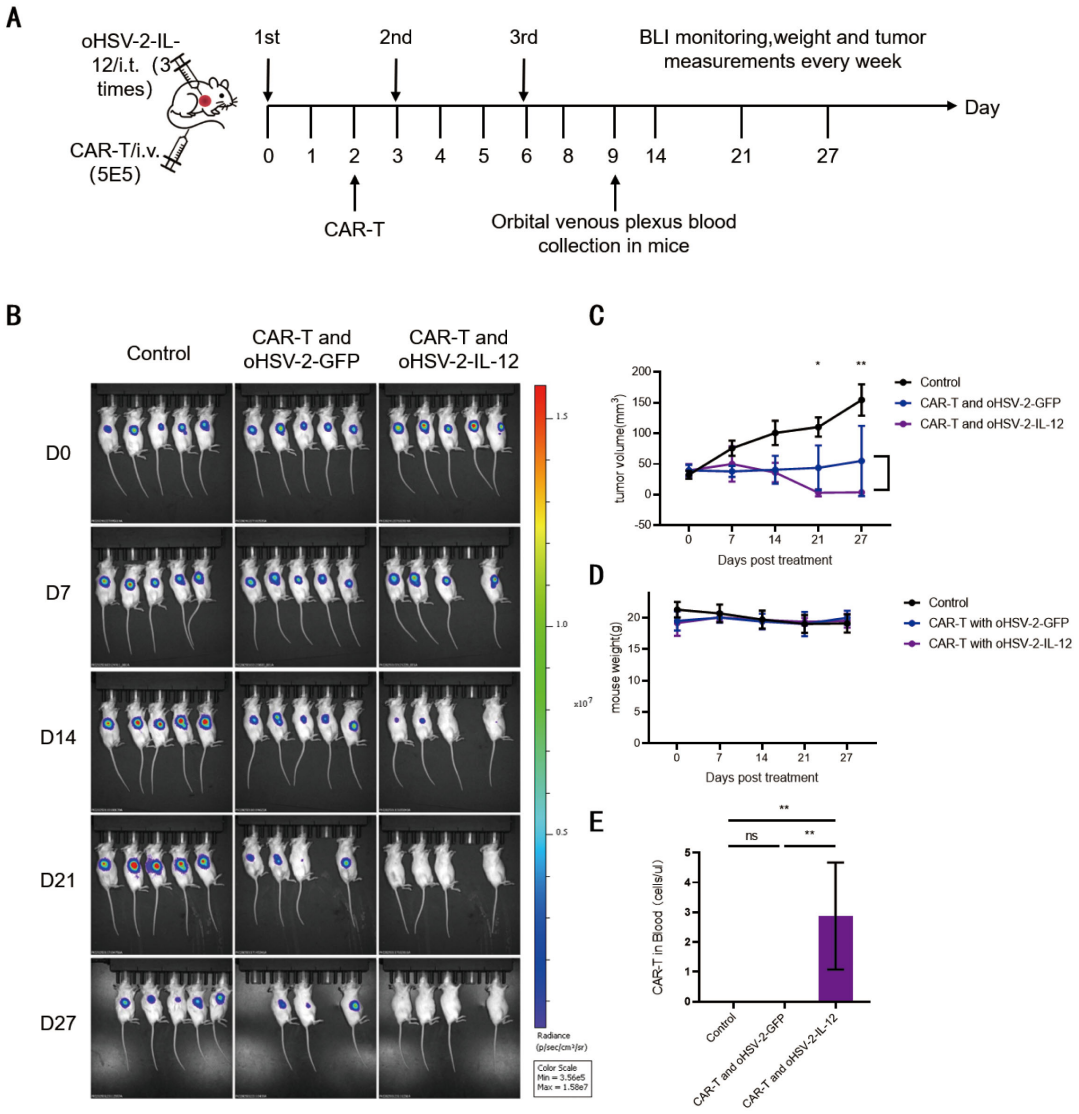
OHSV-2-IL-12 enables complete tumor eradication with reduced CAR-T cell dosage by promoting proliferation of SS1-ICOSBBz-CAR-T cells in peripheral blood of B-NDG mice. **(A)** Schematic of combination therapy with reduced CAR-T dosage and sampling regimen. SS1-ICOSBBz-CAR-T cell dose was reduced to 1/20 (5×10^5^ cells) from 1×10^7^. B-NDG mice (6-week-old) bearing subcutaneous Capan-2-luc tumors (2×10^6^cells implanted 14 days prior) received: intratumoral injections (100 μL) of PBS, oHSV-2-GFP (1×10^5^ CCID_50_), or oHSV-2-IL-12 (1×10^5^ CCID_50_) on days 0, 3, and 6. PBS or SS1-ICOSBBz-CAR-T cells (5×10^5^) were administrated via tail vein injections on day 2. Orbital venous plexus blood collection on day 9. Weekly assessments included bioluminescence imaging (BLI), body weight measurement, and tumor volume caliper measurement. **(B)** Representative weekly *in vivo* BLI images. **(C)** Tumor volume measurements (mean ± SEM; n=5/group). Statistical significance determined by unpaired *t*-test. **(D)** Body weight tracking (mean ± SEM; n=5/group). **(E)** Flow cytometric analysis of CAR-T cells in peripheral blood on day 7 post-CAR-T infusion (day 9 post-first OV injection; mean ± SEM; n=5/group). Significance analyzed by one-way ANOVA with Tukey’s multiple comparisons test. ns: not significant (p>0.05); * p<0.05, **p<0.01.

We observed that oHSV-2-IL-12 enhanced CAR-T cell proliferation and persistence in B-NDG pancreatic cells xenograft tumor model, but the combination therapy of oHSV-2-IL-12 with SS1-ICOSBBZ-CAR-T cells did not demonstrate greater efficacy than the oHSV-2-GFP and SS1-ICOSBBZ-CAR-T combination group as shown in BLI and tumor volume (**Figures 3B, C**). We speculate that it may be due to the rapid tumor clearance within approximately 7 days after treatment, which likely limited viral replication and limited IL-12 expression. As a result, the amount and duration of IL-12 exposure were probably insufficient to further enhance CAR-T proliferation under this high-dose condition. To better evaluate the therapeutic advantage of combination with oHSV-2-IL-12, we reduced the SS1-ICOSBBZ-CAR-T cell to one-twentieth of the original dose (from 1×10^7^ to 5×10^5^ cells). The schematic diagram of the experimental procedure was shown in **Figure 5A**. Briefly, following established combination therapy procedures (including tumor inoculation, oHSV-2 intra-tumor injections 1 × 10^5^ CCID_50_ every 3 days) as previously described, a reduced dose of 5 × 10^5^ SS1-ICOSBBZ-CAR-T cells was administered intravenously one day after the initial oHSV-2 injection. BLI results indicated that the control virus and SS1-ICOSBBZ-CAR-T cells combination group did not achieve complete tumor eradication. In contrast, treatment with oHSV-2-IL-12 and CAR-T cells exhibited tumor regression in some tumor-bearing mice as early as day 14. By day 21, all five mice in oHSV-2-IL-12 combination with CAR-T group showed complete tumor clearance. These findings aligned with previous studies demonstrating that oHSV-2-IL-12 enhances the antitumor efficacy of SS1-ICOSBBZ-CAR-T cells. Consistent with these findings, a significant difference in tumor volumes treated by oHSV-2-IL-12 and CAR-T was observed between the two groups at treatment 21 day and 27 day, as shown in **Figure 5C**. In all groups, there were no significant differences in mouse body weight, indicating that the combination therapy is safer when CAR-T used lower dose (**Figure 5D**). As previously observed, only the treatment group receiving oHSV-2-IL-12 in combination with CAR-T cells—rather than the group treated with oHSV-2-GFP and CAR-T cells—exhibited detectable CAR^+^ T cells in peripheral blood (**Figure 5E**).

This result allowed us to more clearly observe the enhanced efficacy of the oHSV-2-IL-12 and SS1-ICOSBBZ-CAR-T combination therapy over the oHSV-2-GFP and SS1-ICOSBBZ regimen.

Compared with **Figure1E**, we observed that administering CAR-T cells alone at a dose of 1×10^7^ cells was insufficient to completely eradicate tumors. However, when combined with oHSV-2-IL-12, even a significantly reduced dose of CAR-T cells (one-twentieth of the original amount) achieved complete tumor clearance. This finding underscores the synergistic effect of oHSV-2-IL-12 in enhancing the antitumor efficacy of CAR-T cell therapy.

Occasional mouse deaths were observed in the oHSV-2 plus CAR-T cell combination therapy groups(**Figures 3B**, **5B**), whereas no mortality was detected in the oHSV-2 alone or CAR-T monotherapy groups, it suggests there is a risk in combination OV with CAR-T, while, reduced CAR-T cell dose might be safer.

### 3.6 SS1-ICOSBBZ-CAR-T cells are polyfunctional

**Figure 6:**
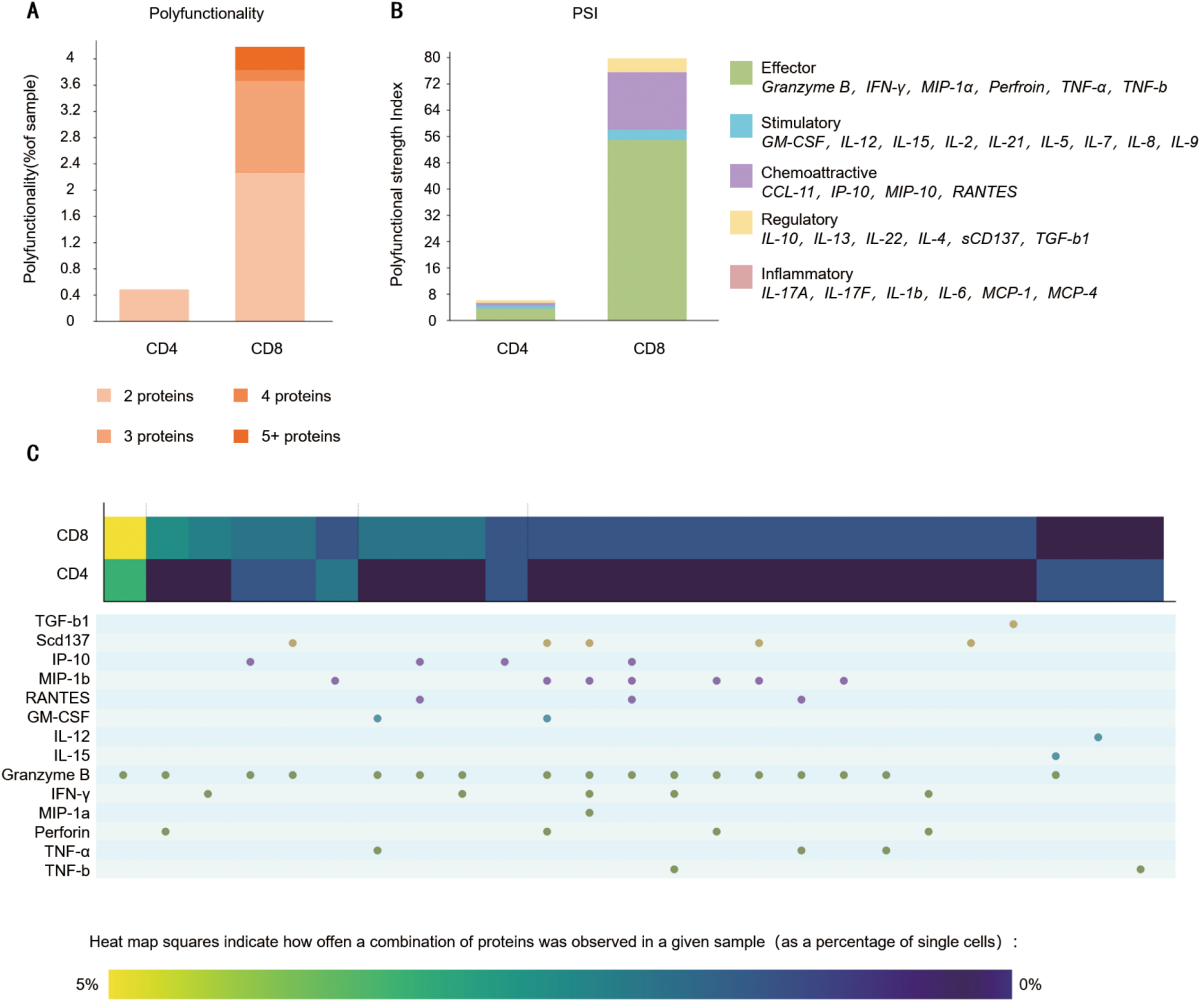
**(A)** Cytokine/chemokine profiles of polyfunctionality of SS1-ICOSBBZ-CAR-T after cocultured with Capan-2-luc. **(A)** polyfunctionality of SS1-ICOSBBZ-CAR-T cells after co-cultured with target cells Capan-2-luc. **(B)** single-cell PSI computed for mesothelin-targeted CAR-T cells co-cultured with Canpan-2 cells for 48 hours at the single-cell level. **(C)** Polyfunctional heat map displaying major functional cytokines/chemokines secreted by CAR-T cells after target cell stimulation. Each column corresponds to a specific cytokine or combination of cytokines, and the red squares represent the frequency at which the group was secreted by the corresponding subsets of CAR-T.

Polyfunctional T cells are recently reported for their association with long-term immune responses in the clinical settings (19).The SS1-ICOSBBZ-CAR-T cells used in our study exhibited moderate tumor-killing activity, the proliferation and persistence *in vivo* were limited. We analyzed their polyfunctional cytokine secretion profiles. Here, we used the 32-plex panel that included the key immune elements of T cells. The experimental results of single-cell secretion of multiple cytokines by CAR-T cells showed approximately 4% of CD8+ cells secreted two or more cytokines, while only 0.4% of CD4+ CAR-T cells were multifunctional, secreting two cytokines, almost no CD4+ CAR-T cells secreted more than two cytokines. As shown in **Figure 6A**, it indicated relatively low multifunctionality in CD4+ cells of SS1-ICOSBBZ-CART. The polyfunctional strength index (PSI) values are defined by cytokine function to highlight the contribution of each group to the overall polyfunctionality of the sample. As shown in **Figure 6B**, CD8+ SS1-ICOSBBZ-CAR-T cells showed 10 times higher than CD4+ CAR T PSI when co-cultured with Canpan-2-luc cells, indicating CD8+ CAR T cells were more polyfunctional than CD4+ CAR T cells. Heatmap analysis, as shown in **Figure 6C**, the most frequently secreted cytokines included Granzyme B, perforin, IFN-γ, IP-10(CXCL11), sCD137(41BB), and GM-CSF. CD8+ cells exhibited a higher secretion frequency of these cytokines. In contrast, CD4+ cells had a lower proportion secreting IL-12, IL-15, and TNF-β in addition to the aforementioned cytokines. These findings further highlight the functional differences between CD8+ and CD4+ SS1-ICOSBBZ-CAR-T cells. The poor proliferation of SS1-ICOSBBZ-CAR-T *in vivo* may be attributed to the low percent multifunctional CD4+ of SS1-ICOSBBZ-CAR-T.

## 4 Discussion

CAR-T cell application to solid tumors faces significant challenges (15). OVs armed with pleiotropic cytokines IL-12 to engage multiple effector mechanisms and reverse tumor-induced immunosuppression, entices cancer researchers (20, 21). In this study, we provided evidence IL-12 expressing oncolytic HSV-2 enhances CAR-T cell efficacy against pancreatic cancer in orthotopic mouse model.

First, as hypothesized, we found that the combination the oHSV-2-IL-12 significantly enhanced the anti-tumor efficacy of SS1-ICOSBBZ-CAR-T cells, leading to complete tumor eradication. This enhanced effect might be attributed to oHSV-2-IL-12 promotion on proliferation and persistence of SS1-ICOSBBZ-CAR-T cells. This combination therapy also facilitated the formation of CAR-T cell-mediated durable antitumor immunity, which prevented tumor formation from re-challenged by Capan-2-luc cells. To elucidate the enhancing effect of oHSV2-IL-12 on CAR-T cells, we employed CAR-T cells with limited efficacy against pancreatic cancer *in vivo*, it is conceivable that utilizing more potent CAR-T cells in combination with oHSV-2-IL-12 could achieve more potent and durable therapeutic benefits.

It is evident that the *in vivo* expansion and persistence of CAR-T cells are crucial for the success of CAR-T therapy (1). We found that SS1-ICOSBBZ-CAR-T cells exhibited poor proliferation *in vivo*, as CD3^+^CAR^+^ cells were undetectable in the peripheral blood following tail vein injection of CAR-T in pancreatic cancer xenograft mouse model. When these CAR-T cells were combined with oHSV2-IL-12, CD3^+^CAR^+^ cells became detectable in the peripheral blood. In contrast, CD3^+^CAR^+^ cells could not be detected in the combination with the control virus oHSV2-GFP, suggesting that IL-12 expression mediated by oHSV-2 infection enhanced CAR-T cell proliferation *in vivo*, leading to substantially improved tumor control. There is a chance that the effect may be attributed to the ICOS co-stimulatory domain, because ICOS can response to IL-12 stimulation and enhanced the effector function of Th (22, 23), or that oHSV-2-IL-12 promotes proliferation may universally in CAR-T cells, because it is also reported that recombinant human IL-12 enhanced activation, proliferation, and cytotoxicity of CEA-CAR-T cells containing a 41BB co-stimulatory domain (without ICOS domain) (24). Recombinant oncolytic adenovirus armed with CCL5, IL-12, and IFN-γ promotes CAR-T infiltration and proliferation *in vivo* to eradicate local and distal tumors (25). These findings warrant investigation into whether oHSV-2-IL-12 similarly enhances other CAR-T efficacy.

IL-12 clearly promotes CAR-T cell proliferation, survival, and cytotoxicity. Mechanistically, IL-12 activates the PI3K/Akt pathway, leading to up-regulation of cell-cycle regulator cyclin D3 and anti-apoptotic proteins including Bcl-2 and c-IAP2, while reducing active caspase-3, thereby supporting T-cell expansion and resistance to apoptosis (26). IL-12 can drive IL-2 independent proliferation of CD4^+^ T cells (27), and engages JAK2/TYK2 and Raf/MEK-1/ERK1/2 signaling to enchance T cell proliferation (28, 29). *In vivo*, IL-12 together with type I interferons prolongs the division of activated CD8^+^ T cells. *In vitro*, IL-12 in conjunction with dendritic cells has been shown to enhance antiviral and antitumor CD8^+^ cytotoxic T-lymphocyte (CTL) responses. Collectively, these documented mechanisms provide a strong literature-supported rationale for oHSV-2-IL-12 enhanced proliferation, persistence, and cytotoxic function of CAR-T cells observed in our study.

In addition to enhancing CAR-T proliferation and persistence in combination therapy, IL-12 also exhibits antitumor activity. We observed a significant reduction in tumor volume in the oHSV-2-IL-12 monotherapy group versus the oHSV-2-GFP control, demonstrating that IL-12-armed oncolytic virus has enhanced antitumor efficacy even in severely immunodeficient B-NDG mouse model. It is reported IL-12 also can recruit and reinforce macrophage function (30, 31). In fact, besides the potent immune-activating capabilities of IL-12 (11), our finding is particularly notable that IL-12 may bolster antitumor responses through modulation of the host’s innate immunity. Therefore, oHSV-IL-12 exhibits enhanced antitumor efficacy, might also be attributed to IL-12’s similar ability to modulate innate immunity.

Notably, our findings revealed that the combination therapy achieved complete tumor clearance while utilizing only 5% of the standard CAR-T cell dosage. In our study, a total cell dose of 1×10^7^ cells per mouse was administered, corresponding to approximately 1.2×10^7^ CAR+ cells/kg for clinical dose based on 23% CAR+ cell frequency. When combined with IL-12-expressing oHSV2, the dose was reduced to 6×10^5^ cells/kg. Compared to the Phase II clinical trial dosages of anti-MSLN-CD28-CAR-T (6×10^7^ cells/kg) combined with pembrolizumab and claudin 18.2-targeted CAR-T (2.5×10^8^ cells/kg) for gastrointestinal cancers (3, 28), our data demonstrate that oHSV2-IL-12 enhances antitumor efficacy while reducing the required cell dose. This represents a substantially lower dose than typical clinical CAR-T regimens. Since the SS1-ICOSBBZ-CAR-T used in this study exhibited suboptimal potency, employing CAR-T cells with enhanced cytotoxicity and proliferative capacity could enable further dose reduction. The optimized dosing result demonstrated significant translational potential. This discovery has significant implications for both manufacturing efficiency and clinical practice. Specifically, lower doses can reduce production costs, and shorten production times, thus enhancing drug accessibility. Clinically, reducing the CAR-T cell dose may decrease the incidence and severity of cytokine release syndrome (CRS), thereby improving treatment safety. Together, these advantages of oHSV2-IL-12 enhance cell adoptive therapy treatment viability and expand patient access.

We observed significant variability in antitumor efficacy among SS1-ICOSBBZ -CAR-T cell products manufactured in different batches, with the causes remaining unclear. The structure is the same as reported by Sonia Guedan et al. (4). But unlike Sonia reported that the CAR-T can completely eradicate pancreatic cancer and exhibit durable antitumor effects, however, as shown in our study, SS1-ICOSBBZ-CAR-T cells alone were insufficient to achieve complete tumor eradication. This suggests that factors beyond CAR structure (32), such as T cell source, manufacturing process, treatment strategies, cancer models and *in vivo* conditions—may influence therapeutic outcomes. Investigating the reasons for varying antitumor efficacy among CAR-T cells with the same construct is crucial for optimizing manufacture conditions or improving therapeutic potency. We hypothesize that poor proliferation and persistence ability of SS1-ICOSBBz-CAR-T cells may be associated with a low proportion of CD4^+^ T cells(37.6%) and their cytokine secretion capacity. Compared to the GPC3-targeting CAR-T reported by Li therapies for liver cancer in mouse tumor xenograft models (19), the CAR-T cells used in our study exhibit similar characteristics in terms of both PSI and polyfunctionality. However, GPC3-targeting CAR-T demonstrated superior survival capability *in vivo*. Therefore, further investigation is still needed to determine the reasons for the poor *in vivo* proliferation ability of our SS1-ICOSBBZ- CAR-T cells.

While IL-12 is known to potently activate immune responses, there are numerous designs of fourth-generation CAR-T cells expressing IL-12. IL-12-secreting CAR-T cells target MSLN show limited antitumor efficacy in preclinical studies, failing to achieve complete tumor clearance while exhibiting increased exhaustion markers (10). Combination with immune checkpoint inhibitors may improve outcomes. Although we utilized different CAR-T constructs and tumor models in our study, the combination strategy with oHSV-2-IL-12 may potentially enhance anti-tumor efficacy. A comparative study between fourth-generation CAR-T therapy and oncolytic virus combination therapy could help determine which approach is more safer, efficient, and cost-effective.

IL-12 secreted by OVs -infected tumor cells significantly promoted proliferation and persistence of CAR-T cells *in vitro* and *in vivo*. The integration of oncolytic virotherapy presents a compelling avenue to enhance CAR-T cell performance. The occasional mouse deaths observed in the combination therapy groups suggest a potential safety concern associated with oHSV-2 and CAR-T combination strategies, possibly related to enhanced systemic inflammation, viral dissemination during rapid tumor lysis, or GVHD-like reactions in CAR-T humanized immunodeficient mice. These findings underscore the need for future studies to systematically evaluate the safety of such combination regimens, particularly in the setting of allogeneic CAR-T therapies such as universal CAR-T, where immune-related toxicities may be further amplified.

It should be noted that the B-NDG mouse model used in this study is severely immunodeficient, which lacks endogenous NK cells, macrophages, dendritic cells, and adaptive immunity, it restricts evaluation of IL-12–mediated crosstalk between CAR-T cells and the host immune system. So it limits to fully recapitulate IL-12–mediated immune interactions. As highlighted by a recent large-scale meta-analysis, results obtained from immunodeficient tumor models often show limited concordance with clinical outcomes in solid tumors (33). Therefore, future studies should evaluate the efficacy and safety of oHSV2-IL-12 in combination with CAR-T therapy in humanized or immunocompetent mouse models, and ultimately in clinical settings.

In summary, our findings suggest that the combination of oHSV-2 with CAR-T cell therapy holds promise for improving treatment outcomes in solid tumors by enhancing the expansion and tumor suppression activity of CAR-T cells and reducing CAR-T treatment dosage.

## Author’s note

XZ, QL work performed entirely at NIFDC.

## Data Availability

The original contributions presented in the study are included in the article/supplementary material. Further inquiries can be directed to the corresponding authors.
