# Supplementary material for: IL-12-armed oncolytic HSV-2 enhances CAR T cell efficacy against pancreatic cancer in xenografted models
Source: Front Immunol. 2026 Jan 16;16:1664289. doi: 10.3389/fimmu.2025.1664289 (PMC12855546; doi:10.3389/fimmu.2025.1664289)
Supplement: Supplementary file 1 [file Table1.docx]

| CAR+%  Group% | Days post treatment | mouse1  CAR-T cells in mouse retro-orbital venous blood detected by flow cytometry every 3 days starting from treatment day 7 | \| mouse2 \| \| --- \| | mouse3 | \| mouse4 \| \| --- \| | \| mouse5 \| \| --- \| |
| --- | --- | --- | --- | --- | --- | --- | --- | --- | --- |
| No treatment | 7 | 0.00 | 0.00 | 0.00 | 0.06 | 0.62 |
|  | 10 | 0.00 | 0.05 | 0.05 | 0.14 | 0.00 |
|  | 13 | 0.55 |  | 0.11 | 0.35 |  |
|  | 16 | 0.00 |  | 0.14 | 0.06 |  |
|  | 19 | 0.00 |  | 0.05 | 0.85 |  |
|  | 22 | 0.20 |  | 2.93 | 0.17 |  |
|  | 27 | 0.00 |  | 0.00 | 0.00 |  |
| SS1-ICOSBBZ-CAR-T | 7 | 0.29 | 0.16 | 0.17 | 0.91 | 0.04 |
|  | 10 | 0.68 | 0.05 | 0.05 | 0.62 | 0.25 |
|  | 13 | 0.23 | 0.06 | 0.41 |  | 0.27 |
|  | 16 | 0.05 | 0.05 | 0.14 |  | 0.00 |
|  | 19 | 0.15 | 0.11 | 0.10 |  | 0.05 |
|  | 22 | 0.26 | 0.23 | 0.00 |  | 0.00 |
|  | 27 | 0.08 | 0.06 | 0.05 |  | 0.00 |
| SS1-ICOSBBZ-CAR-T+OV-GFP | 7 | 0.28 | 0.13 | 0.43 | 0.49 | 0.31 |
|  | 10 | 0.05 | 0.10 | 0.39 | 0.10 | 0.24 |
|  | 13 | 0.06 | 0.12 | 1.38 | 0.11 | 0.16 |
|  | 16 | 0.00 | 0.07 | 0.29 | 0.00 | 0.23 |
|  | 19 | 0.52 | 0.05 | 0.39 | 0.53 | 0.14 |
|  | 22 | 0.00 |  | 0.21 | 0.00 | 0.74 |
|  | 27 | 0.38 |  | 0.05 | 0.00 | 0.00 |
| SS1-ICOSBBZ-CAR-T+OV-IL-12 | 7 | *0.747481243** | *5.505991652** | 0.43 | *0.990109318** | *0** |
|  | 10 | *0.637050119** | *4.24248795** | 2.07 | *0.320912071** | *0.048614661** |
|  | 13 | *8.045967969** | *456.8555708** | 63.80 | *30.3405709** | *4.728920537** |
|  | 16 | *9.699907051** | *2052.610142** | 84.63 | *21.31613362** | *1.61184661** |
|  | 19 |  | *1291.556194** | 17.87 | *6.766856414** | *2.112629124** |
|  | 22 | *0.22* | / | 11.46 | *1.566114502** | *0.665593505** |
|  | 27 | *0.369571553** | *96.92511635** | 3.48 | *1.077552562** | *0* |
